# The Impact of Laser Beam Power on the Microstructure and Some Mechanical Properties of Laser-Alloyed Inconel^®^600 with WC Particles

**DOI:** 10.3390/ma16072619

**Published:** 2023-03-25

**Authors:** Piotr Dziarski, Natalia Makuch, Michał Kulka

**Affiliations:** Institute of Materials Science and Engineering, Poznan University of Technology, Pl. M. Sklodowskiej-Curie 5, 60-965 Poznan, Poland

**Keywords:** laser surface alloying, tungsten carbide, nickel alloys, hardness, nanomechanical properties, wear resistance

## Abstract

Laser surface alloying with WC particles was used in order to improve the wear resistance of Inconel^®^600-alloy. The applied processing parameters ensured appropriate conditions for the production of composite layers enriched with WC particles that did not melt during the process. As a consequence, the produced layers contained globular shaped WC particles that were well bonded in the matrix. The WC particles were characterized by high hardness *H_IT_* = 31.25 GPa and a high Young’s modulus *E_IT_* = 609.33 GPa determined by nanoindentation. The most important parameter influencing the thickness of the layer, the percentage of WC particles in the layer and the wear resistance of the produced layers was the power of the laser beam. Three values of laser beam power were used: 1.3 kW, 1.56 kW, and 1.82 kW. An increase in the laser beam power resulted in an increased thickness of the layer from 435 µm to 685 µm. Simultaneously, when the power of the laser beam was higher, the depth of re-melting of the substrate material increased. This was the reason for a decrease in the percentage of WC particles in the composite layer. The layer produced by laser beam power of 1.3 kW contained 20.59% of WC particles, whereas the highest power of the laser beam (1.82 kW) ensured a percentage of WC particles of about 9.46%. As a result, the increase in the laser beam power was the reason for the reduction in the wear resistance of the composite layer. The lowest mass wear intensity factor (*I_mw_* = 6.4 mg·cm^−2^·h^−1^) characterized the layer produced at laser beam power of 1.3 kW, and the highest *I_mw_* (18.5 mg·cm^−2^·h^−1^) was obtained for the layer produced with a laser beam power of 1.82 kW. However, all the produced composite layers contained WC particles, which ensured improved wear resistance when compared to the Inconel^®^600-alloy without the layer (*I_mw_* = 60.9 mg·cm^−2^·h^−1^).

## 1. Introduction

Tribological wear is one of the most important factors that shorten the effective life of machine components. Friction between mating elements causes such changes as changes in mass and volume and changes in shape and dimensions. In order to avoid these disadvantageous effects, the material should be adequately protected against friction and wear. Surface modification is a good solution often used to improve the wear resistance of various materials. Unique possibilities to modify the surface of materials can be obtained using laser processing, especially by laser alloying or laser cladding. Laser surface alloying (LSA) allows the production of surface layers with a wide range of microstructures and chemical compositions on various substrates. High cooling rates and high solidification rates ensure the achievement of a fine-grained microstructure containing even metastable phases. Metallic elements (e.g., Cr, Ni, Mo), non-metallic elements (e.g., B, C), and ceramics particles (e.g., TiC, SiC, VC, WC) can be used as alloying material during laser surface alloying [1,2].

Due to their high hardness, ceramic particles are often used as an alloying material in laser processing to improve the hardness and wear resistance of various materials. Among the wide group of ceramic particles, tungsten carbide is often used to increase the wear resistance of aluminum alloys [3,4], titanium alloys [5,6] or steels [7,8,9,10,11,12]. Advantageous properties are obtained when composite layers are created in which the matrix is nickel or its alloy and the WC is the component of alloying material [13,14,15,16,17,18,19]. The selection of Ni-based alloys as a matrix for layers/coatings containing WC as a strengthening phase is justified due to the high wettability of nickel powders and its other advantageous properties, such as high toughness, high heat resistance, and corrosion resistance [14]. Generally, the formation of a WC/Ni composite coating by laser cladding increases the microhardness and wear resistance because of the presence of a hard WC phase and a partial dissolution of WC particles in the Ni matrix [13,14,17]. The increase in the WC content in the WC/Ni composite coating results in a gradual increase in hardness and wear resistance. However, it has been proved that in the case of a WC content between 30% and 60%, the wear resistance of the coating does not show significant changes [14]. The high WC content (60%) in the WC/Inconel^®^718 composite coating produced by laser-directed energy deposition was also disadvantageous due to the formation of macrocracks [15]. This situation was caused by a significant difference in the thermal expansion coefficients of the components (WC and Inconel^®^718). It was found that the increase in laser power from 0.9 to 1.3 kW was accompanied by an increase in height of the deposited coatings. With a laser power of 0.9 kW, the height of the deposited part was 2.5 mm, whereas with a laser power of 1.3 kW, the height was increased by about 53.6%.

Another good candidate for the production of laser-clad Ni-based/WC coatings is Ni60 powder containing chromium (15–20 wt.%) and boron (3–4 wt.%) as the main alloying elements [16,18]. It was observed that the average microhardness of the produced coatings increased with the increase in WC content, from 592 HV for a WC content of 5% to 733 HV for a WC content of 10% [18]. Some literature data [19] indicated that the shape of the WC particles influenced the wear behavior of 15 wt.% WC/Inconel^®^625 coatings produced by the laser melting method. Two morphologies of WC particles were investigated: spherical and non-spherical. The coating produced using the spherical WC particles was characterized by a lower coefficient of friction (0.25) than the coating with non-spherical WC particles (0.3). A significant difference was observed in the case of the calculated mass loss of the specimens. The spherical WC/Inconel^®^625 coating achieved a mass loss of 0.007 g after the wear test, and the non-spherical WC/Inconel^®^625 coating mass loss was 0.053 g.

In the present study, composite layers were produced on Inconel^®^600-alloy using a laser-alloying technique. The strengthening phase was WC particles of a spherical shape. In order to investigate the influence of the amount of WC particles in the composite layer on the wear behavior, three different values of laser beam power were used: 1.3 kW, 1.56 kW, and 1.82 kW. The increasing laser beam power was accompanied by an increasing amount of substrate material in the composite layer and a simultaneous decrease in the amount of WC strengthening particles. The influence of the laser beam power, and thus the influence of the amount of WC particles in the composite layers, on the thickness, hardness and wear resistance of laser-alloyed layers produced on Inconel^®^600-alloy was investigated. Nanomechanical properties of the composite layer produced using a laser beam power of 1.3 kW were also analyzed. The aim of this study was to determine the role of the presence of WC particles on wear behavior. Therefore, the surface topography was also examined. Based on the obtained results, a schematic model of the wear mechanism of composite layers strengthened with spherical WC particles was developed.

## 2. Material and Methods

### 2.1. Materials

Inconel^®^600-alloy with a nominal chemical composition shown in Table 1 was used as the substrate material (alloyed material). Ring-shaped samples (external diameter of 20 mm, internal diameter of 12 mm, and height of 12 mm) were used. The alloying material was a paste prepared from the powders of WC (particles size 250–400 µm) and Inconel^®^625-alloy (particles size 50–150 µm). The binder was polyvinyl alcohol. The nominal chemical compositions of the alloying components are presented in Table 1. The desire to not significantly affect the chemical composition and microstructure of the matrix of the composite laser-alloyed layer was the reason for choosing Inconel^®^625-alloy as a component of the alloying material.

### 2.2. Laser Alloying

Laser surface alloying (LSA) by re-melting was applied to produce composite layers enriched with WC particles. This process required the preparation of the alloying material in the form of a paste. Two components were used to prepare the paste: powders of alloying materials (WC, Inconel^®^625-alloy) and binder (polyvinyl alcohol). The weight ratio of the powders was 1:1. Firstly, a paste containing alloying elements was deposited on the cleaned surface of alloyed material (Inconel^®^600-alloy). The thickness of the paste was 240 µm. Only the external cylindrical surface was coated with the paste.

**Figure 1:**
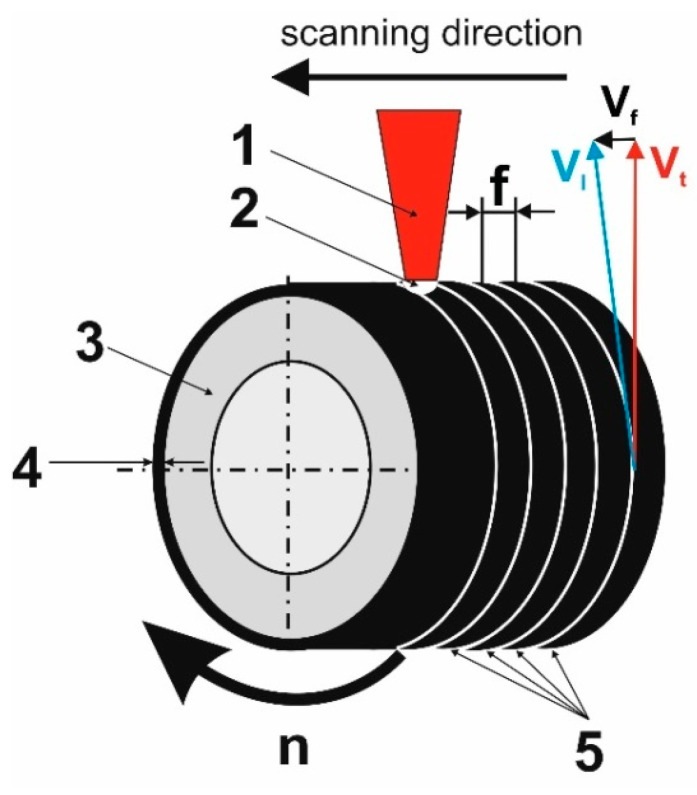
The scheme of laser-alloying by re-melting: 1—laser beam; 2—molten pool; 3—specimen (Inconel^®^600-alloy); 4—pre-coated paste with alloying material; 5—the multiple laser tracks; *v_f_*—feed rate; *v*_l_—scanning rate; *v_t_*—tangential speed; *n*—rotational speed; *f*—distance between the axes of adjacent tracks.

The next stage was the re-melting of the deposited paste together with a part of the substrate material using a laser beam. The laser beam action caused the heating of the sample, and when the temperature exceeded the melting point of Inconel^®^ alloy, the liquid matrix was strongly mixed with the WC particles in the molten pool. A schematic representation of laser surface alloying by re-melting is shown in Figure 1. The process was carried out using a TLF 2600 Turbo CO_2_ laser (TRUMPF, Poznan, Poland), coupled with turning lathe that enabled rotation of the specimens and feed motion of the focusing head, and operated with the following parameters: laser beam power (*P*) 1.3 kW, 1.56 kW or 1.82 kW, scanning rate (*v_l_*) 2.88 m/min, laser beam diameter (*d*) 2 mm, feed rate (*v_f_*) 0.28 mm per revolution, and rotational speed (*n*) 45.85 min^−1^. A multiple-mode laser beam (TEM_01*_) of circular shape was applied. The laser-alloyed layers were produced as multiple laser tracks with a distance between the axes of the adjacent tracks of 0.28 mm (Figure 1). The value of *f* resulted directly from the feed rate *v_f_* (0.28 mm per revolution), whereas scanning rate *v_l_* was determined as a resultant value of feed rate *v_f_* and tangential speed *v_t_* (Figure 1) which was calculated based on the rotational speed of the specimen *n* and external diameter of the specimen (20 mm).

### 2.3. Microstructure Observations and Property Characterization

**Figure 2:**
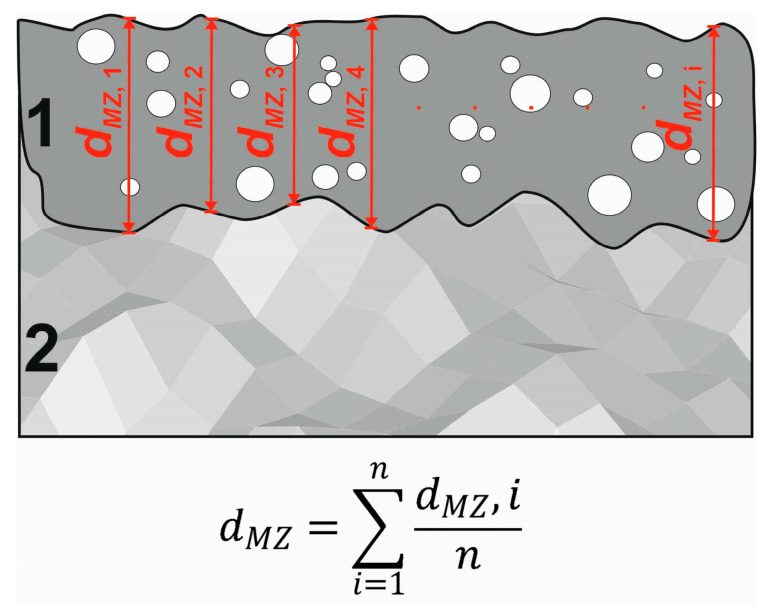
The measurements and calculation of the average thickness of laser-alloyed layer; 1—laser-alloyed layer (re-melted zone); 2—substrate material.

The observations of the microstructure were performed using an optical microscope (OM) LAB-40 (OPTA-TECH. Poznan, Poland) and a scanning electron microscope (SEM) Mira 3 (TESCAN, Poznan, Poland) equipped with an Energy Dispersive Spectrometer (EDS). For investigations, the laser-alloyed samples were first cut across the produced layers in the direction perpendicular to the surface. Such prepared cross-sections were mounted in a conductive resin, ground with SiC abrasive paper, polished with Al_2_O_3_ paste, and then etched with Marble’s reagent. The thicknesses of laser-alloyed layers were calculated as an average value from about 100 measurements carried out in different locations of the cross-section of metallographic samples. In the case of the laser treatment applied, the re-melted zone (MZ) was observed close to the surface, and no changes in the microstructure were noticed below this zone, where the heat-affected zone (HAZ) should occur. Therefore, the depth of the re-melted zone (*d_MZ_*) was assumed to be the thickness of the laser-alloyed layer. The diagram of the measurements and calculation of the average thickness is shown in Figure 2. The surface percentage of WC particles in the microstructure of the layers was determined using binary images prepared on the basis of OM images of the cross-sections of the layers.

Due to the presence of high-hardness ceramic particles (WC) in the matrix of laser-alloyed layers, and simultaneously due to the specific mass transfer in the molten pool during laser treatment, a 3D image of the surface topography of the layer produced at a laser beam power of 1.3 kW was determined. The observations were carried out using a VHX7000 digital microscope (KEYENCE, Poznan, Poland).

In order to prepare microhardness profiles, measurements were carried out along a straight line perpendicular to the surface at different distances from the surface. Tests were performed across the laser-alloyed layers using a Micromet II hardness tester (BUEHLER, Poznan, Poland) equipped with a Vickers diamond indenter under a load of 25 gf (0.245 N).

Nanomechanical properties were determined for the laser-alloyed layer produced with a laser beam power of 1.3 kW. Five measurements were carried out in the WC particles, in the matrix of the laser-alloyed layer, in the boundary between the WC particle and the matrix of layer, and in the substrate material. An NHT^3^ tester (ANTON PAAR. Poznan, Poland) equipped with a Berkovich diamond tip was used for this study. Mechanical properties (indentation hardness *H_IT_* and indentation Young’s modulus *E_IT_*) were estimated according to Oliver and Pharr’s method [20,21]. A maximum indentation load of 50 mN, loading rate of 100 mN/min, unloading rate of 100 mN/min, and pause under a maximal load of 5 s, were used during the measurements.

Dry sliding wear resistance tests were carried out using a friction pair that consisted of an immobile counter-specimen and a mobile specimen. The plate-shaped counter-specimen was made of S20S sintered carbide (dimensions: 12 mm × 12 mm × 5 mm). During the 2 h test, the sample rotated at a speed of 250 min^−1^. The applied load was 149 N and the counter-specimen was changed every half hour of the test. During the test, the mass of the specimen and the counter-specimen were measured every half hour. These measurements were necessary to determine the mass wear intensity factor *I_mw_*, expressed as the mass loss Δ*m* per friction surface *S* and unit of friction time *t* according to the equation:

(1) $$I_{mw}=\frac{∆m}{S\cdot t}$$

where: Δ*m*—mass loss (mg), *S*—friction surface (cm^2^), *t*—friction time (h).

The second parameter describing the wear resistance was the relative mass loss Δ*m/m_i_*, which can be defined as a mass loss Δ*m* in relation to the initial mass *m_i_*:

(2) $$\frac{∆m}{m_{i}}=\frac{m_{i}-m_{f}}{m_{i}}$$

where: Δ*m*—mass loss (mg), *m_i_*—initial mass of specimen or counter-specimen (mg), *m_f_*—final mass of specimen or counter-specimen (mg).

## 3. Results and Discussion

### 3.1. Microstructure of Laser-Alloyed Layers

**Figure 3:**
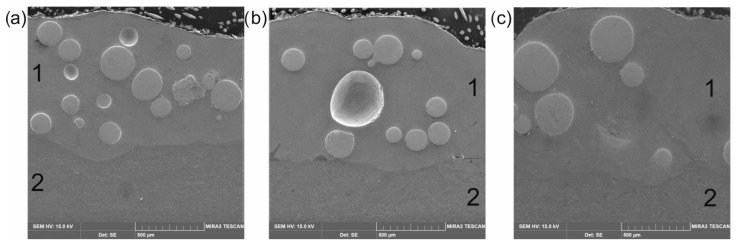
SE images of the microstructure of Inconel^®^600-alloy after laser surface alloying using a laser beam power of: (**a**) 1.3 kW, (**b**) 1.56 kW, (**c**) 1.82 kW. 1—laser-alloyed layer (re-melted zone); 2—substrate material.

**Figure 4:**
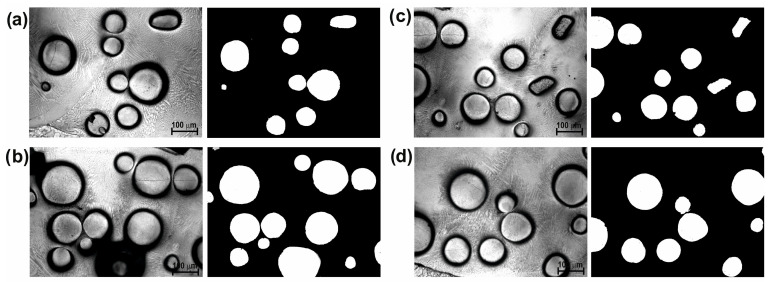
Four selected OM images and their binary images showing the microstructure of the laser-alloyed layer produced with a laser beam power of 1.3 kW: (**a**) area 1, (**b**) area 2, (**c**) area 3, (**d**) area 4.

**Figure 5:**
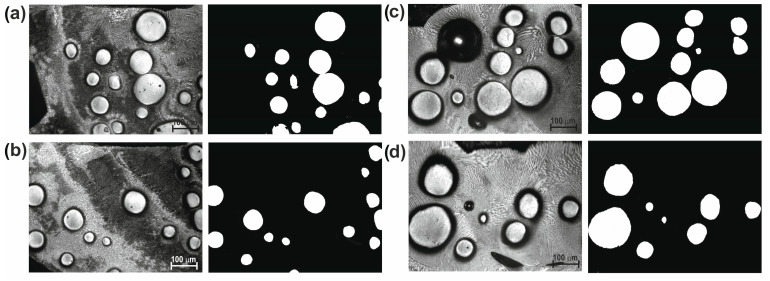
Four selected OM images and their binary images showing the microstructure of the laser-alloyed layer produced with a laser beam power of 1.56 kW: (**a**) area 1, (**b**) area 2, (**c**) area 3, (**d**) area 4.

**Figure 6:**
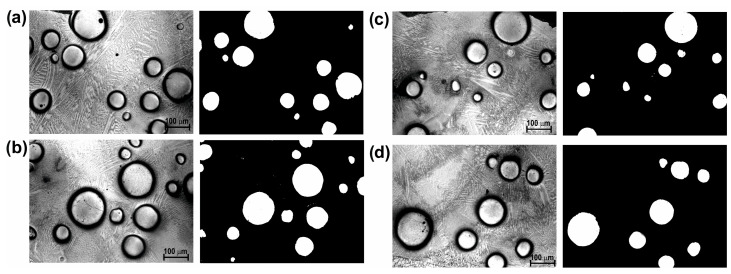
Four selected OM images and their binary images showing the microstructure of the laser-alloyed layer produced with a laser beam power of 1.82 kW: (**a**) area 1, (**b**) area 2, (**c**) area 3, (**d**) area 4.

Figure 3 shows the cross-sectional SEM microscopic morphology of laser-alloyed layers produced using a laser beam power of 1.3 kW, 1.56 kW, and 1.82 kW. All the layers produced were free from porosity and cracks. The spherical WC particles were clearly visible in the produced layers, indicating that the WC particles had not melted during the laser treatment. It was observed that the composite layers enriched with WC particles differed depending on the laser beam power used. The increase in the laser beam power from 1.3 kW to 1.82 kW was accompanied by an increase in the average thickness of the laser-alloyed layer from 435 µm to 685 µm. The laser beam power of 1.56 kW resulted in average layer thickness of 554 μm. The higher power of the laser beam corresponded to the higher temperature generated at a greater depth in the substrate material. Therefore, a larger volume of the melted substrate (Inconel^®^600) was mixed in the molten pool together with the alloying material. It was obvious that the depth of re-melting of the substrate material was dependent on the depth at which the melting point of the substrate material was exceeded (1354 °C). After the laser treatment finished, the produced laser-alloyed layers were visible as multiple laser tracks, the boundary of which could be determined by the line corresponding to the melting point of the substrate material. The laser beam power used during treatment influenced not only the thickness of the produced laser-alloyed layers, but also influenced the amount of WC particles in the composite layer. It was important that, regardless of the sample, the thickness of the pre-coated paste and the amount of WC particles introduced into the molten pool were the same. However, the amount of the substrate material changed, which resulted in a higher volume of the matrix in the layer when using the higher power of the laser beam. As it is known that the amount of strengthening WC particles co-determines the wear resistance, this amount had to be determined. The binary images of layers were used for this study (Figure 4, Figure 5 and Figure 6). First, four selected areas were registered using an OM microscope, and then their binary images were prepared. On the basis of binary images, the percentage of surface area of WC particles per observed area of the cross-sections of the layers was determined. The results are presented in Table 2. As expected, the increase in laser beam power resulted in a decrease in the percentage of WC particles in the laser-alloyed layer. The layer produced with a laser beam power of 1.3 kW contained an average of 20.59% of WC particles. Increasing the power to 1.56 kW reduced the average percentage of WC particles to 15.52%, while the highest power of the laser beam (1.82 kW) provided a WC particle content of approximately 9.46%.

**Figure 7:**
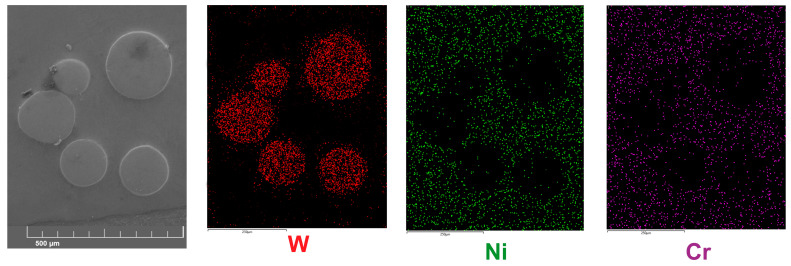
SE image and EDS mapping of tungsten, nickel, and chromium performed for a laser-alloyed layer produced with a laser beam power of 1.3 kW.

**Figure 8:**
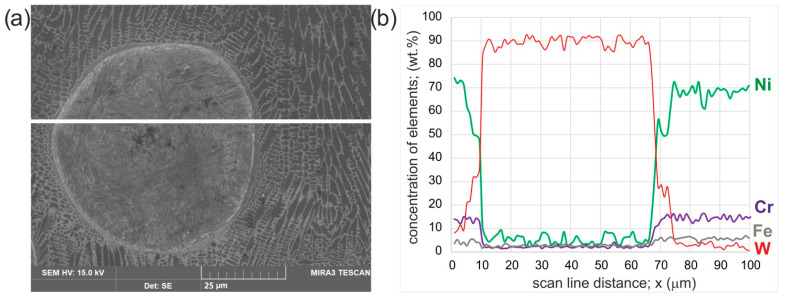
SE image and results of linear X-ray microanalysis performed for a laser-alloyed layer produced with a laser beam power of 1.3 kW; the straight line across a single WC particle (**a**); the concentration of elements across this line (**b**).

The presence of WC particles was detected for all the layers produced (Figure 2). To ensure that the decomposition of these strengthening phases did not occur during the laser re-melting, EDS mapping was performed for the layer produced with a laser beam power of 1.3 kW (Figure 7). It was observed that the WC particles were characterized by an increased concentration of tungsten. While in the matrix, no elevated concentration of this element was detected. However, a slight amount of tungsten was detected in some areas near the interface between the WC particles and the matrix. To confirm this phenomenon, a linear EDS microanalysis was performed along a straight line across a single WC particle (Figure 8). It was clearly visible that the WC particle was characterized by a high concentration of tungsten. On the boundary between the WC particle and the matrix of the laser-alloyed layer, the region with an increased concentration of tungsten (25–28 wt.%) appeared. In this region, a reduced concentration of nickel (50–57 wt.%) was observed compared to the matrix of the laser-alloyed layer. Such a profile of tungsten concentration indicates partial decomposition of the tungsten carbide during the laser beam action. This phenomenon was also observed in the case of high-speed laser cladding of a WC/Ni-based coating [14].

**Figure 9:**
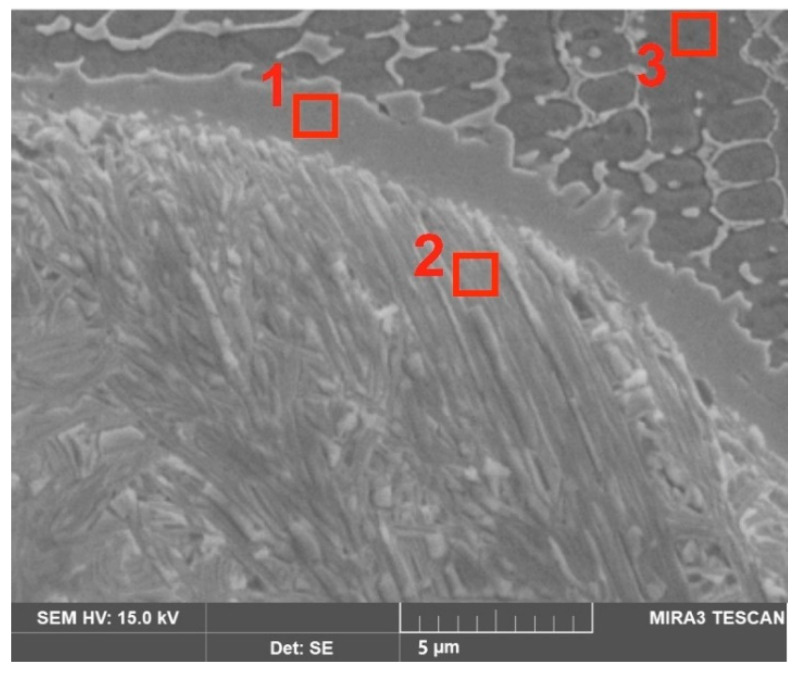
SE image of laser-alloyed layer produced with a laser beam power of 1.3 kW with marked areas in which EDS microanalysis was performed; 1—WC particle; 2—WC/matrix boundary; 3—matrix.

In order to ensure that the region between the WC particle and the matrix of the laser-alloyed layer contains an increased concentration of tungsten, the EDS microanalysis was performed in three characteristic areas of the examined layer (Figure 9). As expected, in the WC particle (area marked as 2) a high concentration of tungsten (92.5 wt.%) was observed. Simultaneously, very low concentrations of nickel (4.2 wt.%), chromium (2.1 wt.%) and iron (1.2 wt.%) were obtained. EDS microanalysis is not preferred to measure the concentration of light elements (such as e.g., carbon, nitrogen), therefore carbon concentration was not investigated. In the area marked as 1 in Figure 9, the following chemical composition was obtained: tungsten (32.2 wt.%), nickel (54.2 wt.%), chromium (12.1 wt.%) and iron (1.5 wt.%). Area 3 in Figure 9 was characterized by a high nickel concentration (72.3 wt.%) and a low tungsten concentration (2.1 wt.%), with simultaneous Cr (17.9 wt.%) and Fe (7.7 wt.%) concentrations characteristic of the Inconel^®^600-alloy. The results of the EDS microanalysis provided two important pieces of information: the spherical WC was only partially decomposed at the edge of the particle; the diffusion zone was formed as a result of diffusion of tungsten atoms from the WC particle to the boundary between the WC particle and the matrix of the laser-alloyed layer.

### 3.2. Microhardness Profiles

**Figure 10:**
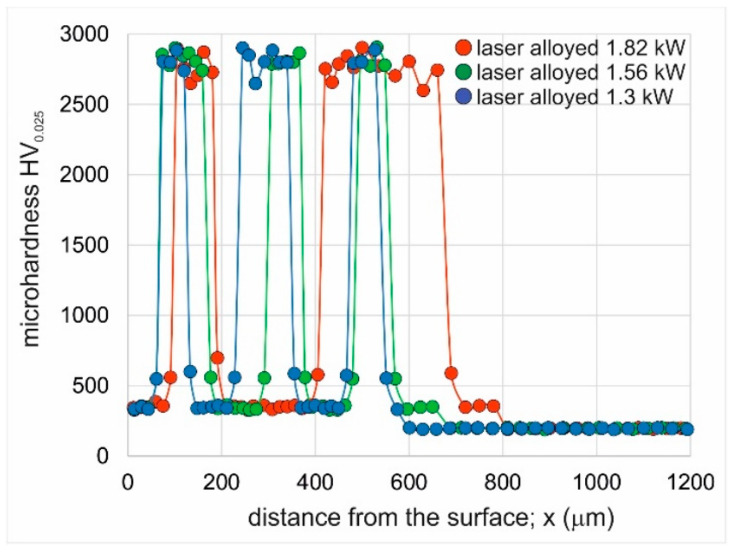
Microhardness profiles of Inconel^®^600-alloy after laser surface alloying with WC particles/Inconel^®^ 625 using a laser beam power of 1.3 kW, 1.56 kW, and 1.82 kW.

The microhardness profiles of the produced laser-alloyed layers are presented in Figure 10. In all the profiles, the local high hardness occurred due to the presence of the undecomposed WC particles in the laser-alloyed layer. The measured hardness on the cross-section of WC particles was in the range of 2600–2900 HV_0.025_. The applied laser beam power had no effect on the hardness of the WC particles, probably because during the laser treatment these particles did not melt. It was noticed that the region at the boundary between the WC particle and the matrix of the laser-alloyed layer was characterized by increased hardness (548–690 HV_0.025_) compared to the matrix of the layer (333–360 HV_0.025_). This situation was caused by the increased concentration of tungsten in this region, as well as by the fine-grained microstructure. Some Vickers indents were performed below the laser-alloyed layer in the substrate material. In such a case, the hardness was about 210 HV_0.025_. The differences between the hardness of the matrix of the laser-alloyed layer and the substrate material require explanation. First, the substrate material was Inconel^®^600-alloy, which differed in chemical composition when compared to the Inconel^®^625-alloy used as the component of alloying material during laser treatment. Second, the microstructure of the matrix that solidified from the molten pool was characterized by a fine-grained dendritic microstructure.

### 3.3. Nanomechanical Properties

**Figure 11:**
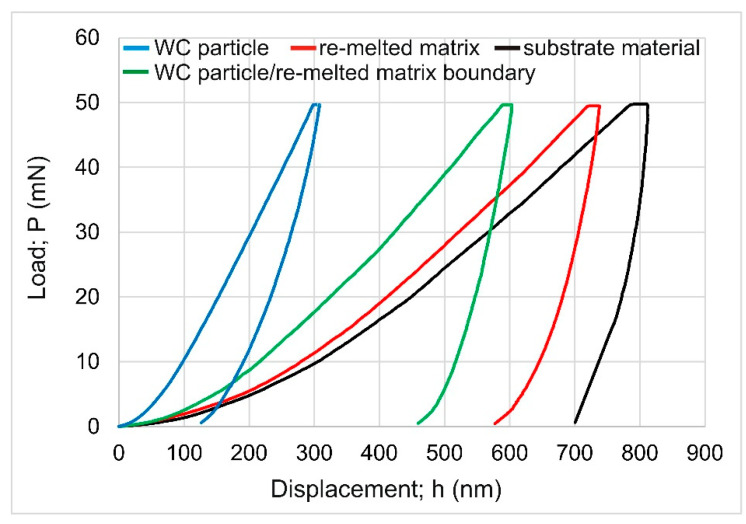
Load-displacement curves recorded for Inconel^®^600-alloy laser-alloyed with WC particles/Inconel^®^625 using a laser beam power of 1.3 kW.

Nanomechanical properties (indentation hardness and indentation Young’s modulus) were estimated based on the load-displacement curves. To calculate indentation moduli (*E_IT_*), the appropriate values of the Poisson ratio had to be assumed. For the substrate material and the re-melted matrix, the average value *ν_s_* = 0.316 was taken based on the literature data [22] concerning Inconel^®^625-alloy (*ν_s_* = 0.314–0.318). In the case of WC particles, the Poisson ratio *ν_s_* = 0.194 was assumed [23]. The selected representative curves recorded for the WC particle, WC particle/matrix boundary, matrix, and substrate material are shown in Figure 11. The curve representing the WC particle was significantly different from the others presented in Figure 10. First, the maximum penetration depth was the lowest (*h_max_* = 309 nm). As a result, the highest indentation hardness (*H_IT_* = 33.79 GPa) and the highest indentation Young’s modulus (*E_IT_* = 598.81 GPa) were measured for the WC particle. Second, the shape of the unloading part of the curve was similar to the loading part, which indicates the highest resistance to indentation among all the tested areas. The average indentation hardness and Young’s modulus measured in the WC particle were 31.25 GPa and 609.33 GPa, respectively. The lowest average values of *H_IT_* (2.85 GPa) and *E_IT_* (216.45 GPa) were obtained in the substrate material (Inconel^®^600-alloy). The differences between the load-displacement curves (Figure 11) indicated that the region at the boundary between the WC particle and the matrix was characterized by higher values for nanomechanical properties compared to the matrix of the laser-alloyed layer. The average indentation hardness and Young’s modulus measured in the region at the WC particle/matrix boundary were 6.89 GPa and 280.29 GPa, respectively. These values were significantly higher than those obtained in the matrix of the laser-alloyed layer (*H_IT_* = 3.95 GPa, *E_IT_* = 233.21 GPa). The reason for these differences was the increased concentration of tungsten in the region at the boundary between the WC particle and the matrix, as well as the fine-grained dendritic microstructure of this area.

### 3.4. Wear Resistance

**Figure 12:**
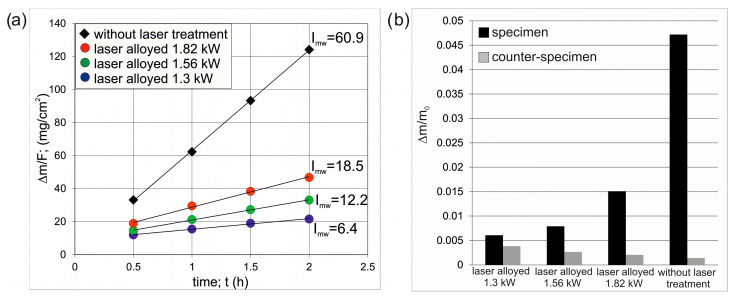
Results of wear resistance tests: (**a**) mass loss of specimens on a unit of friction surface vs. time of friction with calculated *I_mw_* factors, (**b**) relative mass loss of specimens and counter-specimens.

The tribological properties were examined in respect of the mass wear intensity factor *I_mw_* (Figure 12a) and the relative mass loss Δ*m/m_i_* (Figure 12b). The tests were carried out for all laser-alloyed specimens and for the Inconel^®^600-alloy without laser treatment. All produced layers were characterized by a lower factor of mass wear intensity compared to the Inconel^®^600-alloy without laser treatment. However, there were significant differences between the *I_mw_* values for the laser-alloyed samples. The lowest mass wear intensity factor (6.4 mg·cm^−2^·h^−1^) was characteristic of the specimen laser-alloyed with a laser beam power of 1.3 kW. The increase in laser beam power was the reason for the increase in the *I_mw_* value: up to 12.2 mg·cm^−2^·h^−1^ for a specimen laser-alloyed with a laser beam power of 1.56 kW, and up to 18.5 mg·cm^−2^·h^−1^ for a specimen laser-alloyed with a laser beam power of 1.82 kW. These differences resulted from the percentage of WC particles in the laser-alloyed layers. The decrease in the amount of hard WC particles in the layer was accompanied by an increase in the mass wear intensity factor. The highest wear resistance, corresponding to the lowest *I_mw_* value, was characteristic of the laser-alloyed specimen with a laser beam power of 1.3 kW, for which the average percentage of WC particles in the layer was 20.59%. The calculated values of relative mass loss (Figure 12b) confirmed that the layer produced with the lowest laser beam power achieved the highest wear resistance, because the relative mass loss for this specimen was only 0.006078. Increasing the power of the laser beam used during the laser treatment was the reason for obtaining the higher values of Δ*m/m_i_*: 0.007894 for the laser beam power of 1.56 kW and 0.015073 for the laser beam power of 1.82 kW. The lowest wear resistance expressed by the relative mass loss was obtained in the specimen without laser treatment (Δ*m/m_i_* = 0.047174). Interesting observations resulted from the analysis of Δ*m/m_i_* values calculated for counter-specimens. Here the tendency was reversed, i.e., if the tested specimen obtained the lowest relative mass loss then its friction pair counter-specimen obtained the highest relative mass loss. This situation was related to the participation of WC particles in the laser-alloyed layer. WC particles of a high hardness were responsible for the formation of deep grooves and scratches on the surface of the counter-specimen. The higher the percentage of WC particles in the layer, the greater the degradation of the counter-specimen surface. In the case of the layer produced with the use of a laser beam power of 1.3 kW, a high percentage of WC particles in the layer caused a very high wear of the counter-specimen. The relative mass loss of this counter-specimen (Δ*m/m_i_* = 0.003851) was almost as high as that of the tested specimen with the produced layer (Δ*m/m_i_* = 0.006078).

### 3.5. The Specifics of Wear Behavior

**Figure 13:**
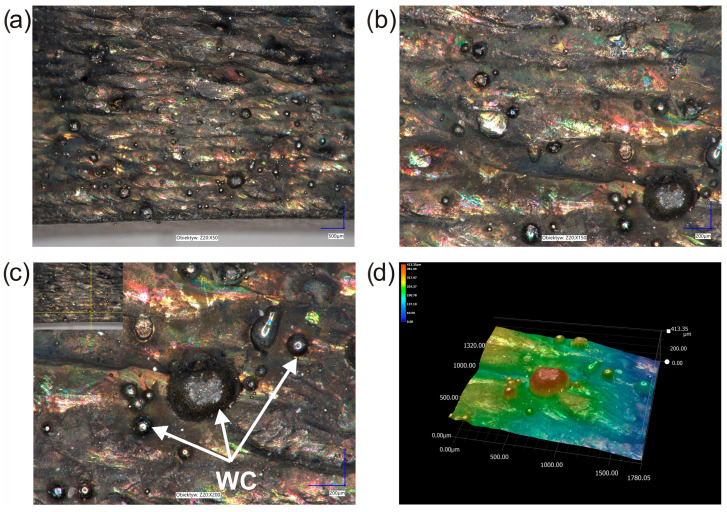
The surface morphology of the specimen laser-alloyed with a laser beam power of 1.3 kW: (**a**–**c**) images recorded by the digital microscope at various magnifications, (**d**) 3D image of the surface topography; the arrows indicate WC particles.

**Figure 14:**
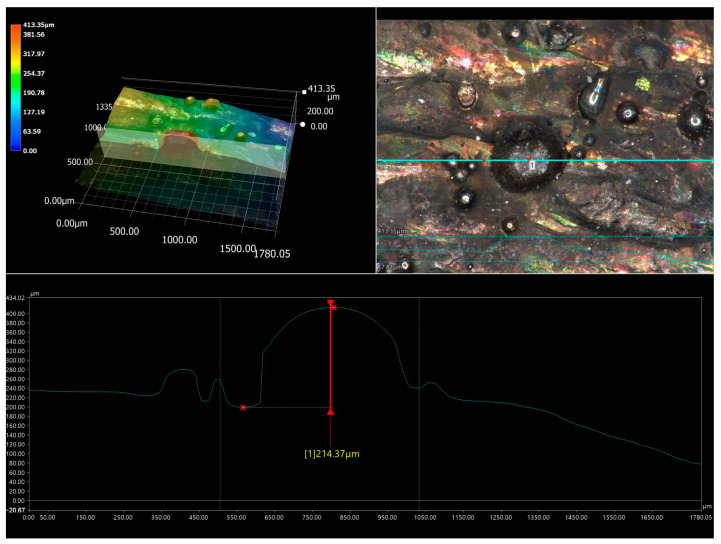
The cross-section of the 3D image of the surface topography and the 2D profile across the WC particle for the specimen laser-alloyed with a laser beam power of 1.3 kW; 1—measuring point of the relative height of the WC particle.

The phenomenon of high wear of counter-specimen used in the friction pair with laser-alloyed specimens require explanation, especially in the case of the layer produced at a laser beam power of 1.3 kW. Therefore, an analysis of the surface topography was carried out. The investigations were performed before and after the wear test for the specimen laser-alloyed with a laser beam power of 1.3 kW. Before the test, the surface of laser-alloyed Inconel^®^600-alloy was characterized by the presence of WC particles (Figure 13a–c). Analysis of the 3D image of the surface topography (Figure 13d) indicated that these particles protruded above the surface of the specimen. To prove that the surface morphology was not flat and the WC particles protruded much higher than the nominal surface of the specimen, the cross-section of the 3D image of the surface topography and the 2D profile across the WC particle were analyzed (Figure 14). The relative height of the largest tungsten carbide particle observed was analyzed and it was found that it reached a height of 214 µm above the surface. Taking into account the diameter of this carbide of approximately 400 µm, it was found that half of it was bound in the matrix of the layer and half of it protruded above the surface. In the case of WC particles with smaller dimensions or in the case of a deeper bonding in the matrix of the layer, the height of the part of the tungsten carbide particle protruding above the surface was lower. The amount of WC particles protruding above the surface of the specimen is a critical factor in both greater wear resistance of the specimen and greater wear of the counter-specimen. In order to explain the role of WC particles in the wear mechanism, an analysis of the surface topography of the specimen after the wear resistance test was performed.

**Figure 15:**
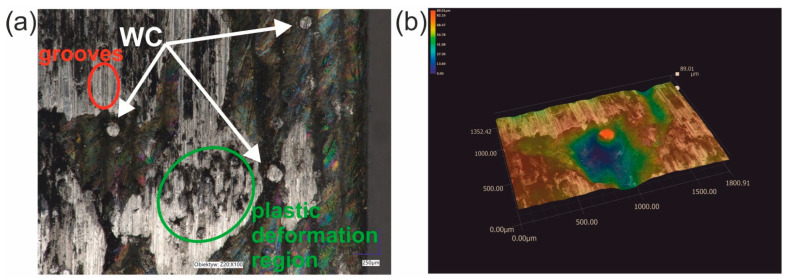
The surface morphology of the specimen laser-alloyed with a laser beam power of 1.3 kW after the wear resistance test: (**a**) image recorded by the digital microscope, (**b**) 3D image of the surface topography; the arrows indicate WC particles.

**Figure 16:**
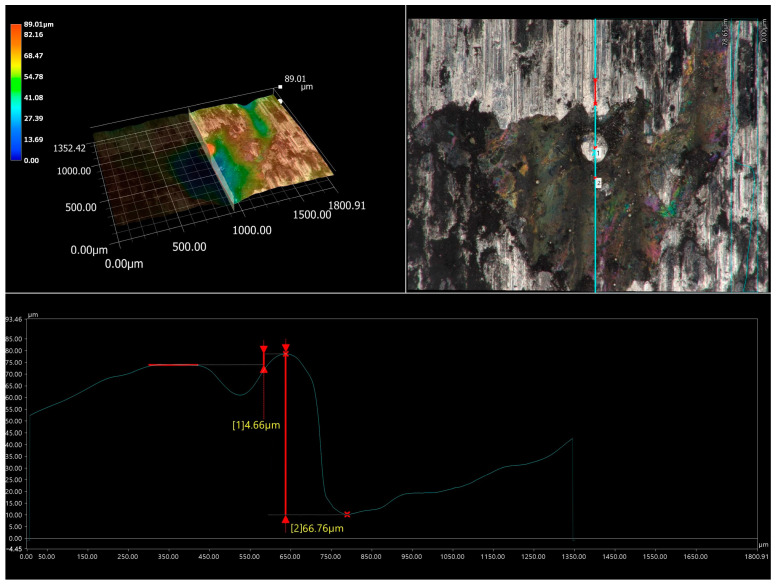
The cross-section of the 3D image of the surface topography and the 2D profile across the WC particle after the wear resistance test performed for the specimen laser-alloyed with a laser beam power of 1.3 kW; 1, 2—measuring points of the relative height of the WC particle.

After the wear resistance test, the surface of the laser-alloyed Inconel^®^600-alloy was characterized by the presence of visible signs of abrasive wear (Figure 15a). Smooth grooves were clearly visible in some areas of the worn surface of the laser-alloyed specimen (Figure 15a). This confirmed the presence of abrasion during the test. However, the abrasive wear mechanism was not the only mechanism occurring during the wear. In some areas the signs of plastic deformation occurred. Moreover, in areas where the plastically deformed surface was visible, small particles pressed into this surface were also noticeable. The presence of such particles was also observed in the case of WC/Inconel^®^625 coatings produced by laser melting deposition [19]. These particles were found to have resulted from the breaking of some of the carbides during the wear test. Therefore, in the present study, it was concluded that the presence of fine particles pressed into the plastic deformation region resulted from the breaking of WC particles during the wear resistance test under a high load of 149 N. It is important to note that that not all WC particles were broken during the wear resistance test. In Figure 14, three tungsten carbide particles were still visible. When the 3D image of the surface topography (Figure 15b) was analyzed, it was clearly seen that these particles still protruded above the nominal surface. The height of the representative WC particle was estimated from a cross-section of the 3D image of the surface topography and a 2D profile across the WC particle (Figure 16). These results confirmed that the WC particles that did not break during the wear test are still higher than the nominal surface of the specimen. Obviously, the WC particles that protruded above the surface of the specimen at the beginning of the wear test play an important role during the wear test.

**Figure 17:**
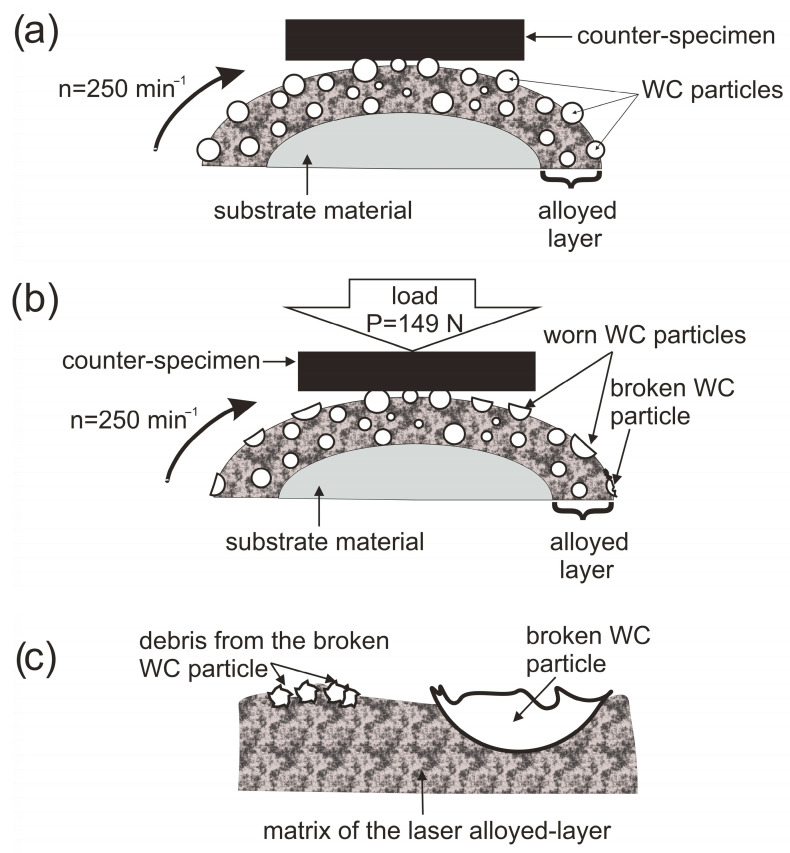
Schematic model of the wear behavior of the laser-alloyed layer produced on Inconel^®^600-alloy: (**a**) the contact between the specimen and counter-specimen, (**b**) first stage of wear, (**c**) details of the area with a broken WC particle and debris pressed into the matrix of the layer.

The role of the presence of WC particles for the wear behavior of the laser-alloyed Inconel^®^600-alloy can be explained using the model shown in Figure 17. It was proved that before the wear test, some of the WC particles protruded above the nominal surface of the specimen (Figure 14). These particles were the first to come into direct contact with the surface of the counter-specimen (Figure 17a). Therefore, at the beginning of wear, the friction pair consisted of WC particles and a counter-specimen. As a result, in the initial stage of wear, these particles were worn and smoothed (Figure 17b), while the matrix of the laser-alloyed layer was protected against wear. Obviously, as the test time passes, the surface irregularities (WC particles) are more and more abraded, which ultimately leads to the exposure of the matrix of the layer. This matrix was characterized by a significantly lower hardness (333–360 HV_0.025_) than the WC particles (2600–2900 HV_0.025_). The exposed low-hardness matrix of the laser-alloyed layer was not only subject to abrasive wear in the form of grooves but was also prone to plastic deformation (Figure 15a). Moreover, due to the low hardness of the layer matrix, debris from broken WC particles could be easily pressed into it (Figure 17c). These debris play an important role in further protecting the layer matrix from wear, but that is not their only role. It is possible that due to the high temperature generated during the non-lubricated contact of the friction pair, the broken WC particles will decompose into tungsten and carbon. As a result of diffusion, these elements can form a new type of phase with the surface of the specimen, e.g., carbides W_2_C, (W, Cr, Ni)_23_C_6_ thus improving the hardness and wear resistance of the layer matrix [19].

**Figure 18:**
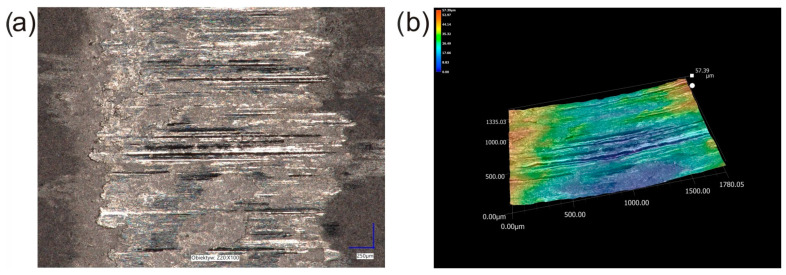
The surface morphology of the counter-specimen mating with the specimen laser-alloyed with a laser beam power of 1.3 kW: (**a**) image recorded by the digital microscope, (**b**) 3D image of the surface topography.

**Figure 19:**
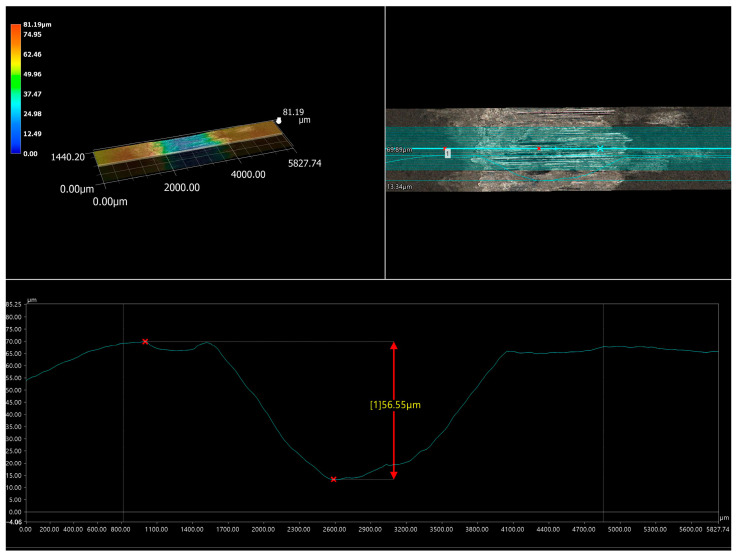
The cross-section of the 3D image of the surface topography and the 2D profile across the worn track formed on the surface of the counter-specimen mating with specimen laser-alloyed with a laser beam power of 1.3 kW; 1—measuring point of the depth of the worn track in relation to the nominal surface of the counter-specimen.

**Figure 20:**
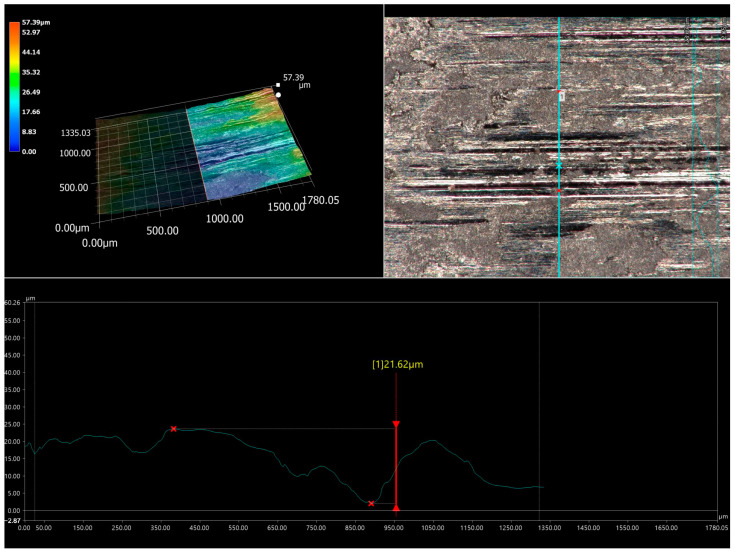
The cross-section of the 3D image of the surface topography and the 2D profile across the grooves formed on the surface of the counter-specimen mating with specimen laser-alloyed with a laser beam power of 1.3 kW; 1 – measuring point of the depth of the representative groove.

The specific behavior of the WC particles during the wear resistance test caused a high relative mass loss of the counter-specimen mating with the specimen laser-alloyed with a laser beam power of 1.3 kW (Figure 12b). The analysis was performed after the first half hour of the test as the counter-specimen was changed every half hour. The worn surface of the counter-specimen was characterized by the presence of deep grooves (Figure 18a). Analysis of the worn track of this counter-specimen (Figure 18b) provided information about its width and shape. The average width of the worn track was 2.1 mm, and the shape of this track was related to the shape of the mating specimen. The ring-shaped specimen caused the worn track formed on the surface of the counter-specimen to be a negative of this shape (Figure 19). The maximum depth of the worn track in relation to the nominal surface of the counter-specimen was 56 µm. The presence of deep grooves on the worn surface of the counter-specimen was caused by the WC particles protruding above the surface of the mating specimen. The cross-section of the 3D image of the surface topography and the 2D profile across the grooves formed on the surface of the counter-specimen mating with specimen laser-alloyed with a laser beam power of 1.3 kW are presented in Figure 20. The maximum depth of the representative groove was 21.62 µm.

## 4. Summary and Conclusions

Laser surface alloying by re-melting was used to produce layers enriched with WC particles on the surface of Inconel^®^600-alloy. The produced layers were investigated in respect of their microstructure, percentage of WC particles, microhardness, nanomechanical properties, and wear resistance. Based on the detailed analysis of the obtained results, the following conclusions could be formulated:

(1) The laser beam power used during laser treatment influenced the thickness of the produced layers. The increase in the laser beam power from 1.3 kW to 1.82 kW was accompanied by an increase in the average thickness of the laser-alloyed layer from 435 µm to 685 µm.
(2) An increase in laser beam power resulted in a decrease in the percentage of WC particles in the laser-alloyed layer. The layer produced with a laser beam power of 1.3 kW contained an average of 20.59% WC particles. Increasing the power to 1.82 kW provided a WC particle content of approximately 9.46%.
(3) WC particles did not decompose during laser treatment; they were therefore characterized by high hardness (2600–2900 HV_0.025_). Only in the region at the boundary between the WC particle and the matrix of the laser-alloyed layer were there signs of the occurrence of partial decomposition of WC. This was confirmed by the EDS microanalysis and the lower hardness of this region (548–690 HV_0.025_).
(4) WC particles were strengthening phases with high indentation hardness (31.25 GPa) and indentation Young’s modulus (609.33 GPa).
(5) All the produced composite layers contained WC particles, which ensured improved wear resistance compared to the Inconel^®^600-alloy without the produced layer. An increase in laser beam power resulted in a decrease in the wear resistance of the laser-alloyed layers. The lowest mass wear intensity factor (*I_mw_* = 6.4 mg·cm^−2^·h^−1^) characterized the layer produced at a laser beam power of 1.3 kW, and the highest *I_mw_* (18.5 mg·cm^−2^·h^−1^) was obtained for the layer produced with a laser beam power of 1.82 kW.
(6) WC particles of a high hardness were responsible for the formation of deep grooves and scratches on the surface of the counter-specimen. The higher the percentage of WC particles in the layer, the greater the degradation of the counter-specimen surface.
(7) The amount of WC particles is a critical factor in both greater wear resistance of the specimen and greater wear of the counter-specimen.
(8) The wear mechanism of the laser-alloyed layer enriched with WC particles was complex. Due to the protrusion of some WC particles above the nominal surface of the specimen, in the initial stage of wear, these particles were worn and smoothed, while the matrix of the laser-alloyed layer was protected against wear.
(9) Some of the WC particles were broken as a result of a high load used during the wear test. The debris from the broken WC particles was pressed into the low-hardness matrix of the laser-alloyed layer. This phenomenon provided further protection for this matrix against wear.

## Figures and Tables

**Table 1:** Chemical composition of Inconel^®^600-alloy used as a substrate material and WC and Inconel^®^625-alloy powders used as alloying material.

Material	Elements (wt.%)										
	Cr	Fe	Mo	Mn	Si	Cu	S	Nb + Ta	C	Ni	W
Inconel^®^600	14.0–17.0	6.0–10.0	-	≤1.0	≤0.5	≤0.5	≤0.015	-	≤0.15	balance	-
Inconel^®^625 powder	20.0–23.0	≤5.0	8.0–10.8	≤0.5	≤0.5	-	≤0.015	3.25–4.15	≤0.1	balance	-
WC powder	0.023	0.2	-	-	-	-	-	0.2	3.95	-	95.627

**Table 2:** Percentage of WC particles in the laser-alloyed layers enriched with WC particles.

		Laser Beam Power *P* (kW)		
		1.3	1.56	1.82
Area 1	Total region area (µm^2^)	349,140	349,140	349,140
	WC particles’ area (µm^2^)	51,708	52,476	48,146
	Percentage of WC particles	14.81%	15.03%	13.79%
Area 2	Total region area (µm^2^)	349,140	349,140	349,140
	WC particles’ area (µm^2^)	105,964	31,527	13,163
	Percentage of WC particles	30.35%	9.03%	3.77%
Area 3	Total region area (µm^2^)	349,140	349,140	349,140
	WC particles’ area (µm^2^)	58,237	87,180	31,807
	Percentage of WC particles	16.68%	24.97%	9.11%
Area 4	Total region area (µm^2^)	349,140	349,140	349,140
	WC particles’ area (µm^2^)	71,644	45,595	38,964
	Percentage of WC particles	20.52%	13.06%	11.16%
Average percentage of WC particles		20.59%	15.52%	9.46%

## Data Availability

The authors confirm that the data supporting the findings of this study are available within the article.
